# Operation for Cataract

**Published:** 1870-04

**Authors:** 


Novel Operation.—M. Hervey de Chegoin reports, October 13th, to the Imperial Surgical Society a successful operation for cataract upon an ass 21 years of age. The size of the eye necessitated the construction of special instruments, and the large size of the pupil and the softness of the lens permitted the passage of a portion of it into the anterior chamber, from which it soon disappeared by absorption. The operator remarks naively enough, that owing to the difficulty of adopting biconvex lenses, the patient was enabled to pursue his occupation (which was principally eating), without them. — *Ibid.*

